# Freeze-drying platforms design for batch fabrication of Haversian system mimicking scaffolds with enhanced osteogenesis

**DOI:** 10.3389/fbioe.2022.1013528

**Published:** 2022-10-11

**Authors:** Licheng Lin, Xiuhong Huang, Zhentao Li, Guiyin Zhang, Hongbo Yu, Yi Wan, Changren Zhou, Lin Zhou

**Affiliations:** ^1^ Department of Materials Science and Engineering, Engineering Research Center of Artificial Organs and Materials, Jinan University, Guangzhou, China; ^2^ Traumatology Department,The First Affiliated Hospital of Jinan University, Guangzhou, China; ^3^ School of Applied Chemistry and Materials, Zhuhai College of Science and Technology, Zhuhai, China

**Keywords:** Haversian system mimicking scaffolds, freeze-drying platforms, chitosan/collagen type I, osteogenesis, directional freeze

## Abstract

The Haversian system is one of the most important pathways to repair bone defects, and it is the basic guarantee for the repair of bone defects, which means that the formation of the Haversian system indicates repairing of the defects. The integration of structure and function for tissue engineering scaffolds is of great importance in mimicking native bone tissue. However, in contrast to the increasing demands, how to rapidly prepare various sizes of such Haversian system mimicking scaffolds in batch becomes a major challenge. In this study, we designed three types of platforms with different sizes in combination with the freeze-drying approach. Chitosan/type I collagen composite materials were used to study the structure, morphology, and performance of the production, and the effects of the controlled architecture on osteogenesis. Results showed that the physicochemical effects of the mass fabricated scaffolds of various sizes met the requirements of bone repair materials. In addition, the scaffolds had good cytocompatibility and excellent *in vivo* bone repair performance, which have potential clinical applications.

## Introduction

Since the first clinical use of artificial organs in 1940s, biomedical materials have really entered a period of rapid development, and many bone repair materials have shined in the field of bone defects today (Szpalski et al., 2010; Perez et al., 2018). The integration of structure and function for tissue engineering scaffolds is of great importance in mimicking native bone tissue. It is well known that the Haversian system is located between the inner and outer annulus and is the first part of the bone tissue to be generated, providing a lumen for vascularization, neurogenesis, and creating conditions for the transport of various nutrients required for new bone production. Therefore, in the process of repairing and regenerating damaged bone tissue, the construction of a tissue repair scaffold that mimics the Haversian system is very important for osteogenesis, angiogenesis, and neurogenesis, as well as the transport of different types of cells. In general, the larger the animal (including humans), the less the size of the components of the Haversian system is affected by age, location of the skeleton, and so forth. The Haversian canal of primates (including humans) is stable at about 250 μm. Interestingly, 250 μm is the size bottleneck of the Haversian canal, even for animals much larger than humans (e.g., cattle and horses) whose Haversian canal size is around 250 μm. Even within the same organism, the shape of the Haversian canal can take on a variety of elliptical shapes, but in general, the shape of the Haversian system tends to be circular due to the presence of the bone plate. If the Haversian canal is defined as the *Z*-direction, the distribution of bone tubules in the bone plate is centered on the Haversian canal in the XY plane (Jowsey, 1966; Maggiano et al., 2021).

Processing strategies such as freeze-drying, 3D printing (Zhang et al., 2020), and electrospinning (Kim and Lee, 2011) have been utilized to construct the biomimetic Haversian system. However, the focus of the studies has always been on the Haversian canal, and little attention has been paid to the generic nature of the whole system of mimicry and preparation methods, especially for batch processing of the constructs. In fact, the optimal pore size of scaffold materials remains controversial for applications in bone tissue repair. For example, the sizes of 200–400 µm (Zhang et al., 2010), >300 µm (Karageorgiou and Kaplan, 2005), 100–350 µm (Vasita and Katti, 2006; Zhang et al., 2010), and 150–710 µm are almost not different in the effects on osteoblast proliferation, and 400–600 µm is beneficial for new bone formation and vascularization. Smaller pores size leads to limited vascularization and hypoxic conditions, which can induce osteochondral formation (Karageorgiou and Kaplan, 2005). Therefore, having a graded pore size is helpful in forming multiple tissues within a single scaffold and regulating the rate of scaffold dissolution (Karageorgiou and Kaplan, 2005). Pore size and porosity offer many advantages for the materials, but they do decrease the structural strength of the scaffolds. Therefore, the pore size of bone tissue engineering scaffolds needs to be compromised and balanced (Hofmann et al., 2007).

In fact, directional freezing has excellent performance in aspects such as pores orientation, pores diameter control, and mechanical strength of the substrate (Bhattacharjee et al., 2017; Algharaibeh et al., 2019), even benefit for the cryo-polymerization (Kirsebom et al., 2009; Barrow and Zhang, 2013). Currently, directional freeze-casting is commonly used to build and design materials that have certain structural requirements (Wu et al., 2012). These structures are used to enhance damping, elasticity, and fracture toughness of the material. (Chau et al., 2016; Knöller et al., 2018; Huang et al., 2019). To supply a fast but simple and versatile fabrication of the Haversian system with hierarchical orientation of the pores, the key point is to design work platforms for directional freezing. Herein, three types of platforms with different sizes in combination with the freeze-drying approach were designed in this work. In terms of raw materials selection, properties such as certain mechanical strength, biocompatibility (including osteoconductivity and osteoinductivity), and biodegradability (Meinel et al., 2005; Wang et al., 2006; Wenk et al., 2009; Chao et al., 2010; Swetha et al., 2010; Venugopal et al., 2010; Holzwarth and Ma, 2011; Rockwood et al., 2011; Farokhi et al., 2013) were considered; we selected type I collagen and chitosan. Collagen exists in muscles, bones, nerves, and skin tissues and can effectively promote cell adhesion and proliferation. Because of abundant mineral deposition points in the collagen structure, it can induce calcium deposition in bone tissue, but its mechanical properties and plasticity are poor, so it is often used in combination with other materials to further enhance biological activity (Velleman, 1999; Kuppan et al., 2013; Datta et al., 2017; Xu et al., 2019; Li and Zhuang, 2020).

In recent years, there have been many excellent reports on directional freeze-casting (Shahbazi et al., 2020), most of which have focused on inorganic materials (Du et al., 2022) and on how the prepared materials perform (Yin et al., 2018), that is, on why they work. In contrast, we not only focus on why materials can have bone repair effects but also explore how these materials with bone repair effects can be prepared, in terms of structural mechanics, thermodynamics, and so on. After that, we use these principles to design the platform.

The challenge of the study is to construct a universal preparation platform based on the directional freezing technique that mimics the structure of the Haversian system formed in the early stage of bone tissue healing. Cavities of the Haversian system mimicking scaffolds were almost at right angles to each other. On this basis, the bone repair performance of Haversian system mimicking scaffolds was studied more systematically *in vivo* and *in vitro*, and its results showed that the scaffold has good prospects for clinical therapeutic applications.

Herein, inspired by the natural bone structure, a Haversian system mimicking fabrication platform was designed, and the chitosan/collagen composite scaffolds were successfully prepared. The method provided a fast and robust strategy to fabricate structurally diversified scaffolds from a single precursor solution by a one-step process.

## Materials and methods

### Materials

The Haversian system platforms A, B, and C are prepared using copper (Chinese National Standard T1, Oudifu, China), rigidity close area polyurethane (Chinese National Standard: 95% percentage of close area, Chengyue, China), and extruded polystyrene sheets (XPS, Chinese National Standard, Chengyue Building Materials, China). Chitosan (AR 90%, Shanghai Macklin Biochemical Co., Ltd., China), PBS (AR, Guangzhou Chemical Reagent Factory, China), type I collagen (extracted from beef Achilles tendon, Wuxi BIOT Biology Technology Co., Ltd., China), and vacuum freeze drier (Alpha 2-4 LSC, Martin Christ Gefriertrocknungsanlagen GmbH, Germany).

### Design of the platform components

**FIGURE 1:**
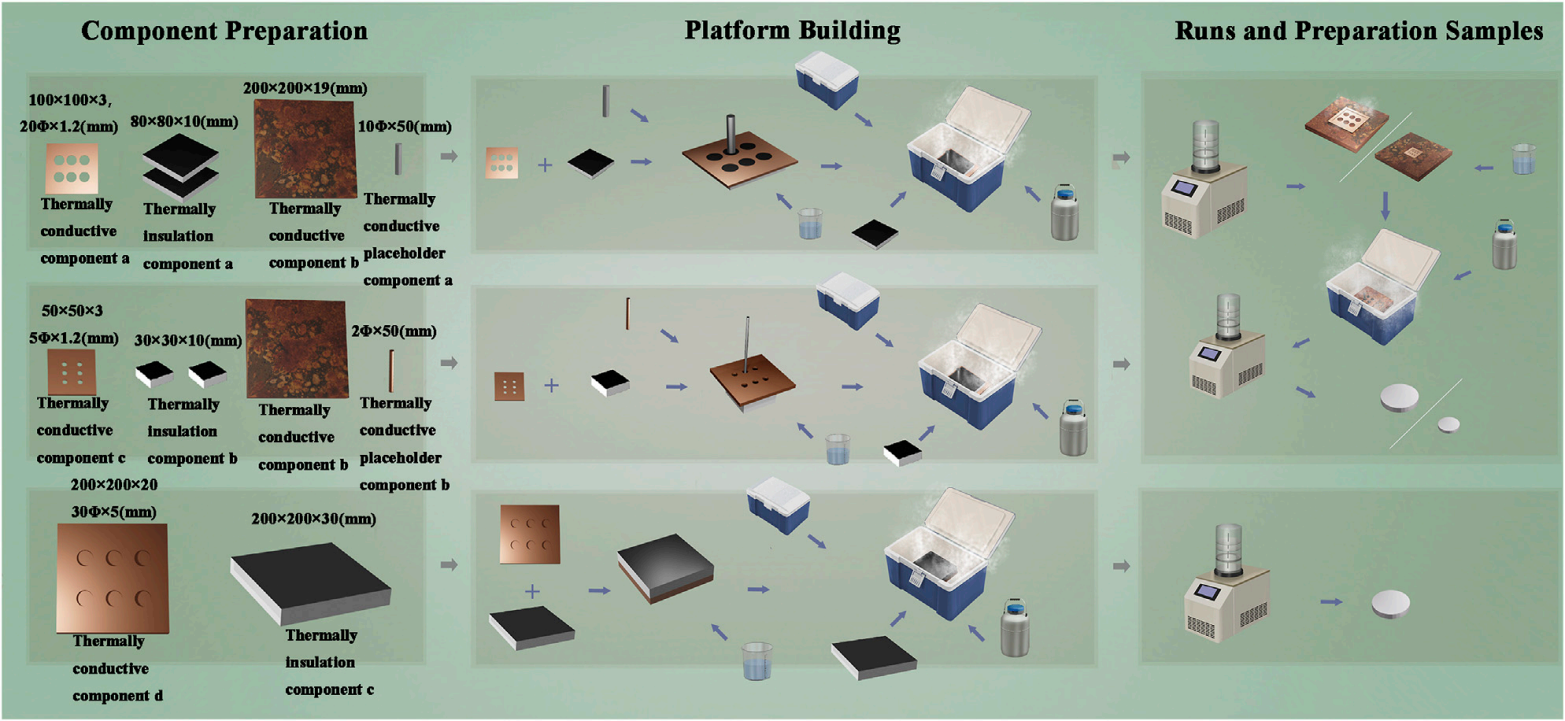
Working schematic diagram of the three types of platforms.

The platform components models were created using AutoCAD 2019, and three types of the platforms were designed. Based on the cranial defect dimensions used in most Sprague Dawley (SD) rats models, platform B was with the size of Φ5 mm × 1.2 mm. Platform A and platform C were designed with a transverse diameter of c. a. 20 mm for the inner condyles and a transverse diameter of c. a. 30 mm for the outer condyles, for the large animal models, as shown in Figure 1. The thickness and diameters of the components can be superimposed to satisfy the different sizes of defects, in line with the overall composition of the human bone Haversian system. The axial superposition of the same type of platforms does not affect the structure of the material mimicking the Haversian system.

The difference in thermal conductivity of the materials results in the rate of thermal radiation transfer from the preform being transformed from a uniform undirected value to a vector value with direction and magnitude. The thermal radiation of the preform is transferred faster in one or more directions, resulting in the ice crystals in that direction being the first to form and occurring of phase separation; the ice crystals will always grow in that direction as they conduct heat faster than the water phase which has not yet phase separated and crystallized. The specific parameters of the preliminary modeling are as follows.

Platform A design data: Thermally conductive component a: overall size 100 mm × 100 mm × 3 mm, six evenly spaced holes, 20 mm diameter, 1.2 mm length, and bottom surface taken out 80 mm × 80 mm × 1.8 mm. Thermally conductive component b: overall volume 200 mm × 200 mm × 19 mm. Thermally insulation component a: overall volume 80 mm × 80 mm × 10 mm and two pieces. Thermally conductive placeholder component a: 10 mm diameter and 50 mm height.

Platform B design data: Thermally conductive component c: overall volume size 50 mm × 50 mm × 3 mm, evenly distributed six holes, hole diameter 5 mm, hole length 1.2 mm, and bottom surface taken out 30 mm × 30 mm × 1.8 mm. Thermally insulation component b: overall volume 30 mm × 30 mm × 10 mm and two pieces. Thermally conductive placeholder component b: overall size 2 mm diameter and 50 mm height.

Platform C design data: Thermally conductive component d: overall volume 200 mm × 200 mm × 20 mm, six perforated holes, 30 mm diameter, and 5 mm hole length. Thermally insulation component c: overall volume 200 mm × 200 mm × 30 mm.

### Preparation of Haversian system mimicking CSCol scaffolds

A volume of 50 ml 4% chitosan (CS) solution was added with 1 and 0.5 g type I collagen, respectively, to obtain CS_4_Col_50_ and CS_4_Col_25_ pre-solutions; 50 ml 2% CS solution was added 0.5 and 0.25 g type I collagen, respectively, to obtain CS_2_Col_50_ and CS_2_Col_25_ pre-solutions.

The experimental solution was fed into the work platforms, and the CS_2_Col_25_ pre-solution was added to a 30-ml screw-top vial. The solution was mixed with a cross-linking agent and kept stirring. The solution was left to stand for 12 h, the pH was adjusted to 7, placed in a −20°C environment for 12 h, and then freeze-dried for 24 h to obtain CS_2_Col_25_ samples. CS_2_Col_50_, CS_4_Col_50_, and CS_4_Col_25_ were used as the CSCol experimental groups and CS_2_Col_25_ as the CSCol control group and placed in a 4°C environment until use. The scaffolds made by different types of the work platforms were named ACSCol, BCSCol, and CCSCol, respectively.

Platform A and B operation as follows. The thermally conductive component a and the thermal insulation component a are assembled into the sample tank component a. The pre-solutions and cross-linking agent are added to the sample tank component a. The sample tank component a and thermally conductive placeholder component a are assembled into the molding component a. The overall ambient temperature of the bio-incubator and thermally conductive component b is adjusted to −50°C using liquid nitrogen. Overall ambient temperature is maintained at this temperature until ready for use. The molding component a is put with the bio-incubator. Then the bio-incubator is closed. After 30 min, the molding component a is removed and freeze-dried for 24 h. The thermal insulation component a and thermally conductive placeholder component a are removed from the molding component a; the thermally conductive component a is kept upside down on the thermally conductive component b, and this forms the molding component b. The molding component b is kept in the bio-thermal adjusted overall ambient temperature to 4°C. Pre-solutions and cross-linking are added to the remaining space. The overall ambient temperature is lowered to −50°C. After 30 min, thermally conductive component a is freeze-dried for 24 h.

Platform C operation as follows. The pre-solutions and cross-linking agent are kept in the hole of thermally conductive component d. The overall ambient temperature of the bio-incubator is adjusted to −50°C using liquid nitrogen. Thermally conductive component d is put with the bio-incubator. Then the bio-incubator is closed. After 30 min, the thermally conductive component d is freeze-dried for 24 h.

### Characterization of the CSCol scaffolds

The scaffold morphology was observed and analyzed by using a scanning electron microscope (SEM Ultra 55, Carl Zeiss AG, Germany), Fourier transform infrared spectrometer (FT-IR), and GT; the porosity of the gel scaffold was tabulated using the liquid displacement method (Lee et al., 1997). Specific parameters are mentioned in the Supplementary Material.

The macroscopic structure of the ACSCol and CCSCol scaffolds was observed by stereomicroscopy. The macroscopic structure of the BCSCol and the microscopic structure of the scaffolds were observed by SEM. The cross-sectional morphology and pores were analyzed.

The mechanical properties of each group of gel scaffolds were analyzed by a stress and strain analysis using electromechanical universal testing machines (AG-Ⅰ, Shimadzu Corporation, Japan). In total, three sets of parallel tests were made for each sample in both directions and averaged. The compression rate was 1 mm/min and the strain was set at 70%/60%.

The porosity of the scaffolds was tabulated using the liquid displacement method. The volume of the gel scaffold was recorded as V, the container full of ethanol was obtained as M1, and the remaining ethanol in the container after the scaffold was immersed was recorded as M2.

### Cytocompatibility of the scaffolds

Cell activity was assessed using acridine orange/ethidium bromide (AO/EB) staining, and at the appropriate time points, AO/EB working solution was added to each well and cell activity was recorded using an inverted fluorescence microscope (Axio ObserverA1, Carl Zeiss AG, Germany) observations. The effect of CSCol on cell proliferation was probed using a Cell Counting Kit-8 (CCK-8). After culturing human umbilical vein endothelial cells (HUVEC) for 1, 3, and 5 days, cell proliferation was detected using the CCK-8 kit, and the optical density (OD) of each well was recorded at 450 nm using a multifunctional microplate reader (Epoch, BIOTEK, United States) (Li et al., 2021; Yu et al., 2022; Wu et al., 2023).

### Bone defect repair *in vivo* with mimicking Haversian system gel scaffolds

SD rats were anesthetized with sodium pentobarbital intraperitoneally; the right leg was shaved at the knee joint to expose the surgical site, and the skin was disinfected with iodine. A 1-cm linear skin incision was made at the distal femoral epiphysis of both hind limbs and the muscle was bluntly dissected to expose the femoral condyles. A 3-mm diameter to 3-mm depth osteotomy perpendicular to the bone axis is drilled at a slow pace using a dental ring drill. After rearing to week 1 (1W)/2W/4W, the rats were anesthetized and the material was taken. The material was processed for hematoxylin-eosin (H&E) staining, Masson’s staining, TRAP staining, immunofluorescence staining, immunohistochemical staining, and micro computed tomography (micro-CT) imaging for observation and evaluation (Huang et al., 2022).

## Results and discussion

### Platform operation and component material selection

The platform is based on bi-directional freezing, which is a step up from directional freezing. The essence of directional freezing technology is to transform the temperature conduction from a three-dimensional non-vector value to an approximate two-dimensional vector value, which requires human control of the temperature conduction speed and direction to form a differential thermal conductivity; bi-directional freezing is to transform the temperature conduction into two or several approximate two-dimensional vector values. The platform design utilizes materials with different rates of thermal conductivity to absorb heat the precast fluid differently at different locations, resulting in bi-directional freezing of the precast fluid and ultimately the preparation of mimicking Haversian system gel scaffolds. The role of the bio-thermal chamber component is to stabilize the low temperature environment around the molding components a, b, c, and d and the thermally conductive component d during platform operation. The thermal conductivity of pure copper is known to be better among common materials, slightly less than precious metals such as pure silver and graphene, which are expensive, and the processing of graphene or graphite is difficult. Pure copper is divided into T1, T2, and T3 licenses according to Chinese national standards, of which T1 copper has the least number of impurities, and fewer impurities can make the thermal conductivity of the material closer to isotropic, which is conducive to the preparation of gels.

After research, high purity copper and most biocompatible materials and their cross-linking agent short contact reaction is not violent after long contact with water, and carbon dioxide and oxygen are easy to form Cu_2_(OH)_2_CO_3_ and Cu(HCO_3_)_2_ because the product is relatively single; the later platform maintenance is easy and efficient and conducive to extending the platform life.

There is a dimensional difference between the thermally conductive components of the three types of platforms, but none of them are welded. The welding process is likely to lead to changes in the density, strength, thermal conductivity, and specific heat capacity of the components and raw materials; the prefabricated liquid is easy to react with the raw materials and lead to corrosion of the components; and the liquid nitrogen quenching process has a great chance of weakening the strength of the welded joints, leading to shortening the service life of the platform and increasing the maintenance cost of the platform.

The platform is a modular solution to the need for rapid fabrication of different parts and different defect repairs. The design of the modules is based on relative thermal conductivity volumes. Module designs are based on relative thermal conductivity volumes and temperature transfer vectors.

The use of materials with low thermal conductivity to control heat transfer can lead to an increase in the vector value of differential thermal conductivity. By the common materials thermal conductivity and national standards in the selection, rigid closed-cell polyurethane and XPS thermal conductivity is low and closed-cell rate is high so that when it receives extrusion it will not immediately expel gas in the material, to protect the thermal insulation performance and its mechanical properties, high closed-cell rate so that the thermal insulation components a will not be infiltrated by the prefabricated liquid, conducive to quantitative prefabricated liquid and waterproof performance. In summary, rigid closed-cell polyurethane and XPS meet the requirements for platform erection due to their processing properties, water resistance, and mechanical properties. Compared to rigid closed-cell polyurethane, XPS is simpler to prepare on the basis of sacrificing certain performance and meets the design purpose of the C-shaped platform, so it was replaced.

The platform component test results compared to Chinese national standards have been placed in support of the information (Supplementary Tables S1,S2).

This chapter only discusses the relevance of the choice of materials for building platform preparation to the principle of oriented freezing, and the principle of gel scaffold preparation will be discussed in conjunction with the analysis of gel scaffold morphology.

### Morphology of the scaffolds

**FIGURE 2:**
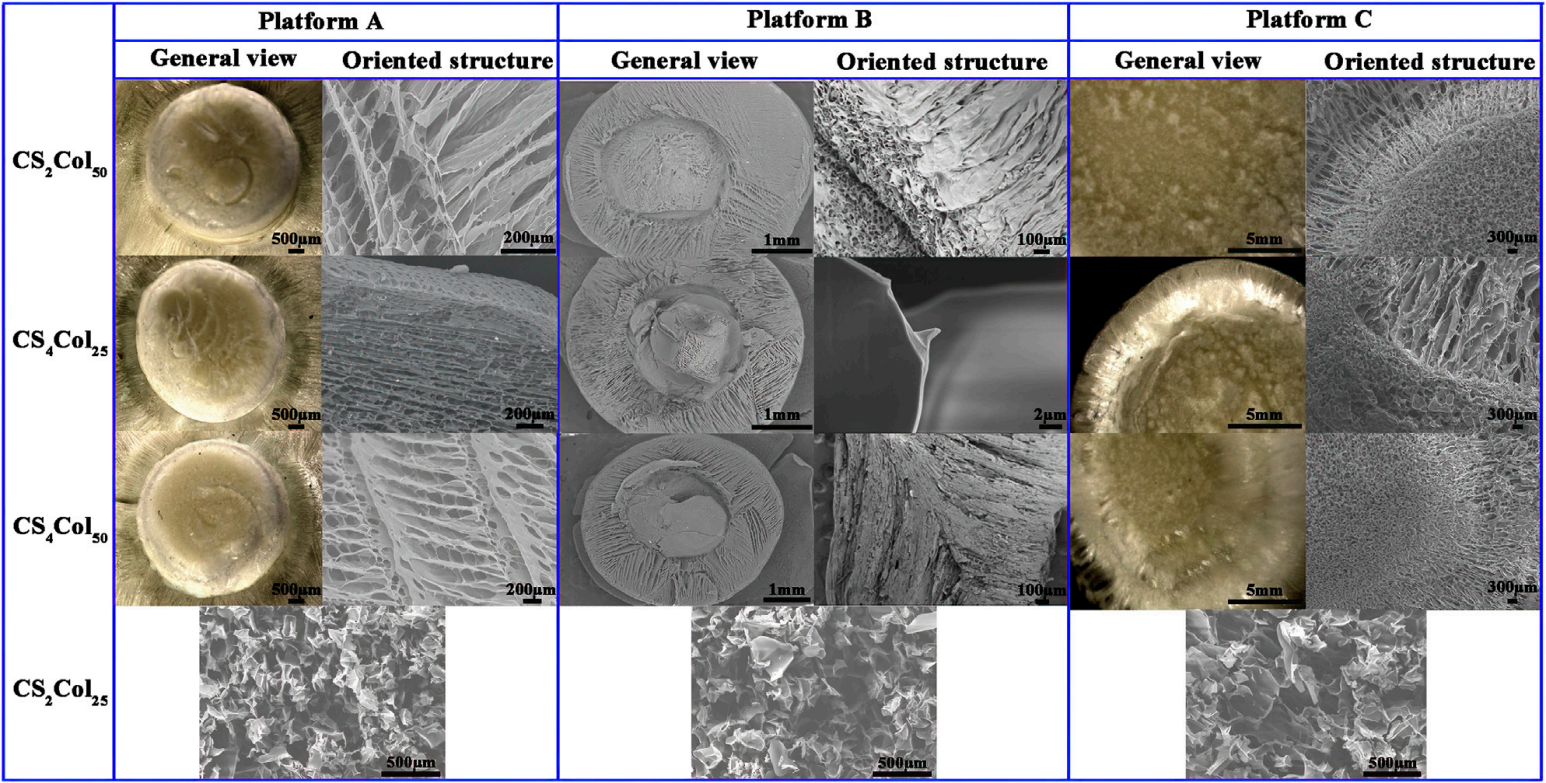
Morphologies of the CSCol scaffolds fabricated by different types of the platforms. (Changed the picture of CCS4Col25, oriented structure of platform A, and general view and oriented structure of platform C; added the picture of ACS2Col25, BCS2Col25, and CCS2Col25).

It was observed that the CS_2_Col_25_ samples had no significant differences in the morphology of the bi-directional cut profiles in the three types of platforms, which are homogeneous, disordered and unoriented structures. Other CSCol scaffolds show arranged bi-directional alignment morphologies, wherein the outer oriented channels radiate from the center core, which forms a longitudinal, highly oriented honeycomb canal. The size of the pores was in the range of 80–300 μm, similar to that of the Haversian Canals in the human Haversian system. Compared with the CS_2_Col_50_, the structures of the CS_4_Col_50_ scaffolds are denser, due to the larger feeding concentration, ice crystal formation and the bi-directional compression force of the sample tank compartment. As can be seen from Figure 2, the walls of the oriented pore channel are smooth, with several smaller holes formed perpendicular to the axial direction of the oriented channel. These holes were created due to the connection between the ice crystals during the ice crystal growth process, as a result of the inevitable volume expansion of the precast liquid during the transformation of the aqueous phase, but as the ice crystals formed by the water in the confined compartment undergo phase separation from the other phases, the ice crystals and the compartment continuously give the other two phases. This results in a very tight structure of the pores walls.

The small holes in the walls of the aligned channels are in fact the “defects” that occurred when the other two phases were unable to resist the two-way compressive stress of the ice crystals and the bulk at low temperatures, and it is these defects that allows the ice on either side to be joined together. These “defects” cause the collapse of the oriented pores purely in terms of the mechanical stability of the materials but have the advantages that the size is both the minimum size of the endothelial cells and the effective transport of nutrients between the oriented pores, so that these “defects” can effectively increase biocompatibility. The small pores, mimic the Volkmann canal of Haversian system, can provide a constant supply of nutrients to the cells in the oriented pores.

The pore lengths were affected by the bi-directional compression forces failing to extend to the top of the material, in fact a very few samples of CS_4_Col_25_ showed a similar situation, while CS_2_Col_50_ of the three types of platform types were the most stable with the least sample variation. No significant interfacial effects were found at any of the interfaces at the junctions of the bi-directional pores, and the spatial connection between the radially oriented pores and the axially oriented pores was good. It can be concluded that an increase in parameters proportional to the bi-directional compressive stress (e.g., raw material stiffness, feeding volume, etc.) will lead to the growth of oriented pores.

The forming mechanism of the three types of platforms was analyzed in conjunction with SEM. After the molding component a is placed in the bio-thermal incubator at a constant temperature, the already cold thermally conductive component b is brought into contact with the thermally conductive component a via a connector, and only 5 min later the temperature of the thermally conductive component a has dropped below 5°C when the liquid nitrogen is added for the first time under the infrared temperature measurement gun, while the thermal insulation component a remains above 10°C. When the incubator is opened for the second time after 10 min and the liquid nitrogen is added, the temperature of the thermally conductive component has dropped to about 1°C, while the thermal insulation component a was still maintained at about 3°C. At this point the low temperature of the thermally conductive component a had started to induce a phase transition in the water, which then caused the ice crystals in contact with the thermally conductive component a to start to grow radially centripetally along the circular hole, and by 15 min the temperature of the thermally conductive component a had dropped to below 0°C, while the thermal insulation component a had also dropped to around 1°C. The temperature of the upper thermal insulation cover was tested at around 5°C, and Thermally conductive placeholder component a was around 1°C. As the ss316 used in the occupancy device was also very fast in conducting heat at 25 min the overall temperature of all components of the molding component a had fallen below 0°C, indicating that the preform had completely completed its phase transition. The freeze-dried ice crystals sublimate leaving radially oriented pores.

Unlike the molding component a, the molding component b has the thermally conductive component b. Due to the relatively large area of the direct contact the thermally conductive component b, the thermal conductivity of the thermally conductive component b is superior by virtue of its larger cross-sectional area and direct contact with the liquid nitrogen, and the effect of differential thermal conductivity can be achieved without pre-cooling. The ice crystals start to grow from the bottom and eventually form axially oriented pores throughout the central part after freeze-drying. The overall temperature change was also slightly different, as the thermally conductive component b was in direct contact with the liquid nitrogen, which resulted in the temperature of the thermally conductive component b dropping to around 3°C by 5 min. The temperature of the gel in the centripetal aperture was then affected by the thermally conductive component a. The temperature of the thermally conductive component a also dropped to around 5°C. At 10 min the temperature of both the thermally conductive component a and the thermally conductive component b reached around 0°C. As the temperature of the sample could not be measured directly, the whole platform was kept in operation for 30 min to ensure that all the water in the sample had completed its phase transition. The gel in the radially centripetal oriented pore section was converted to a hydrogel due to direct contact with a small portion of the prefabricated liquid hydrogel that had not yet been lyophilized, which resulted in the overall gel not showing a significant interface after freeze-dried.

It is worth noting that the temperature of both the molding component a and the molding component b decreases more and more slowly with time, the rate at which the temperature decreases imply the rate at which the aqueous phase separates and freezes, as will be explained in the next paragraph in relation to the phase transition and separation and the generation of oriented pores.

When the two-way compressive stress is applied to the water molecules that have not yet formed ice crystals, the water molecules that have not yet formed ice crystals undergo a relative enthalpy change, and the enthalpy change in thermodynamics is not discussed in depth here. The process of generating ice crystals is an exothermic process, and it is this recurring cycle that makes the temperature drop more and more slowly, but of course this “slow” is a very inaccurate description because the platform environment is around −50°C this “slow” is a relative process, the actual situation is in the it is only at the beginning of the platform’s operation and near −1°C that temperature statistics can be found, and only at the stage near the end of all ice crystal growth is the pressure sufficient to affect the aqueous phase transition temperature, and many experiments have found that at temperatures below −5°C, the temperature of the molding component drops rapidly until it approaches −50°C.

In fact, after several attempts, the 20 mm aperture is already the limit size of the thermally conductive component a. For this platform, exceeding the 20 mm aperture will lead to a radially oriented pores that not only cannot be fully extended to the center as the ice crystal extends further and further away from the cold source and the pressure increases under the influence of the lowering of the freezing point the growth rate of the ice crystal slows down making the volume of the ice crystal larger, making it difficult to ensure the size of the aperture This is one of the limitations of this platform.

In order to achieve faster thermal conductivity, the platform B was reduced from a 20 mm aperture to a 5 mm aperture and maintained a 1.2 mm aperture length compared to the platform A, resulting in a 93.75% reduction in sample volume and a 75% reduction in the thermally conductive component, which is 125% of the relative volume of the thermally conductive component of the platform A. The reason for maintaining the 1.2 mm aperture is that the efficiency of heat radiation is not only related to the specific heat capacity and volume of the heat transfer material but also to the area of contact. A larger volume of material at the same temperature and specific heat capacity will provide or receive more heat radiation, while a larger contact cross-sectional area at the same temperature, specific heat capacity and volume of material will provide faster heat radiation. The reduction of the radial area is due to the conclusion reached during the preparation of the platform A sample that increase in the parameter that makes the pressure rise in both directions (e.g., material stiffness, feeding volume, etc.) leads to the growth and elongation of the oriented pores. As a result of this improvement, the length of the radially oriented pores has increased significantly, the number of penetrated axially oriented pores has increased significantly.

The overall temperature variation is slightly different for the platform B compared to the platform A. The platform B was not found to find the phenomenon that the temperature of the molding component c and the molding component d decreases more and more slowly with the passage of time, which is due to the small volume of the molding component c and the molding component d itself with small heat travel, large relative area, and strong ability to receive thermal radiation from the prefabricated liquid, and the freezing point reduction brought about by enthalpy change has minimal impact.

Compared to the platform A and B the thermally conductive components, the thermally conductive component of the platform C is larger and more difficult to prepare, but still cannot be prepared by the welding process of choice because not only does the density strength change after welding, but welding also has an effect on the thermal conductivity and specific heat capacity, and the welded joints are very susceptible to corrosion. The most fatal problem is that the sudden cooling of liquid nitrogen can significantly weaken the strength of the welded joints and thus shorten the life of the platform and increase maintenance costs. Therefore, the thermally conductive components s a, b, c and d are machined in one piece.

The radial dimensions, axial dimensions, overall dimensional uniformity and overall structure of the gel scaffold orientation orifices prepared for the three types of platforms have proven to be satisfactory. For mixed feedstocks with solvents with freezing points below the temperature of liquid nitrogen, the three types of platforms were able to prepare Haversian system mimicking scaffolds, initially achieving the construction of a platform from an individual case to a universal platform. The length of the access pore is one of the limitations of this set of platforms.

### Mechanical strength testing of gel scaffold

For a qualitative comparison of the “elasticity”, according to the stress and strain curves and the mechanical properties of the material, there are three stages in the compression process: elastic deformation, inhomogeneous plastic deformation, homogeneous plastic deformation. This is defined by looking at the ratio of the deformation to the total deformation when the upper yield point was reached: the larger the value, the more elastic the material.

**FIGURE 3:**
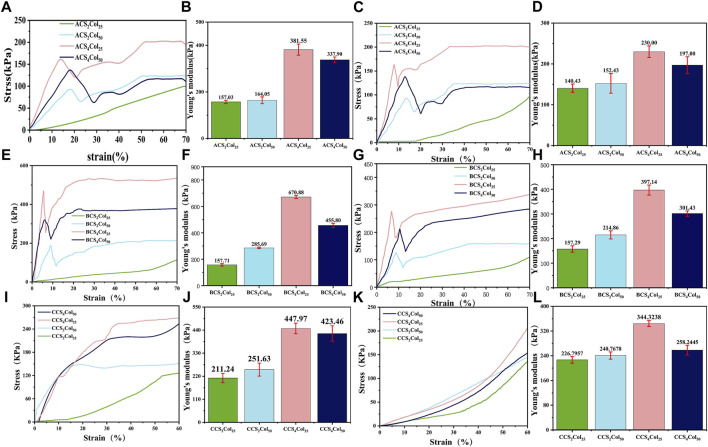
Stress and strain curves and Young’s modulus of the CSCol scaffolds. Longitudinal stress and strain curves and Young’s modulus of the ACSCols **(A,B)**, BCSCols **(E,F),** and CCSCols **(I,J)**. Transverse stress and strain curves and Young’s modulus of the ACSCols **(C,D)**, BCSCols **(G,H)**, and CCSCols **(K,L)** (changed the figure’s color of ACS4Col25/BCS4Col25/CCS4Col25/ACS2Col25/BCS2Col25/CCS2Col25).

As can be seen from Figure 3, the scaffolds exhibit completely different mechanical properties in different direction,fully demonstrating the anisotropic character. Analysis of the stress and strain results for the three types of the CSCol scaffolds in relation to their composition shows that both in the longitudinal and transverse direction, the CSCol experimental groups are all superior in terms of elasticity, 70% compressive strength and strength limit, compared to the normal freeze-dried CS_2_Col_25_ scaffolds. The values in the longitudinal direction are all higher than those in the transverse direction, in some degree.

In terms of 70% compressive strength in combination with Young’s modulus data, the scaffolds fabricated with 4% (weight, wt.) chitosan solution are better than those with 2% (wt) chitosan solution, mainly due to the increased content of chitosan as the main load-bearing materials. With the loss of the central oriented central core structure support, the chitosan content fraction is less susceptible to intrinsic structural defects with lower porosity. A comparison between the two 4% chitosan groups shows that the compressive strength of the CS_4_Col_50_ with higher Col content is lower than that of the CS_4_Col_25_. In terms of the elasticity of the gel scaffold, CS_4_Col_25_ is significantly the stiffest, although it possesses the same collagen concentration with the CS_2_Col_25,_ and the same chitosan concentration with the CS_4_Col_50._ Scaffolds fabricated by the three types of platforms show obvious differences of the strength. BCSCols possess the highest strength and modulus, and CCSCols have the lowest, due to the radial sacrifice of a certain length of oriented pores in platform C.

### Porosity of the scaffolds

The SEM characterization showed that almost all the pores in the void structure of the bionanogel scaffold were larger than 500 nm, in which case the nitrogen uptake and desorption method was not suitable. Therefore, the liquid displacement method was chosen to characterize the porosity of nanofiber membranes in this topic, calculated as follows:

$$P=\frac{M_{1}-M_{2}}{V\rho_{ethanol}}\times 100\%.$$ (1)

**FIGURE 4:**
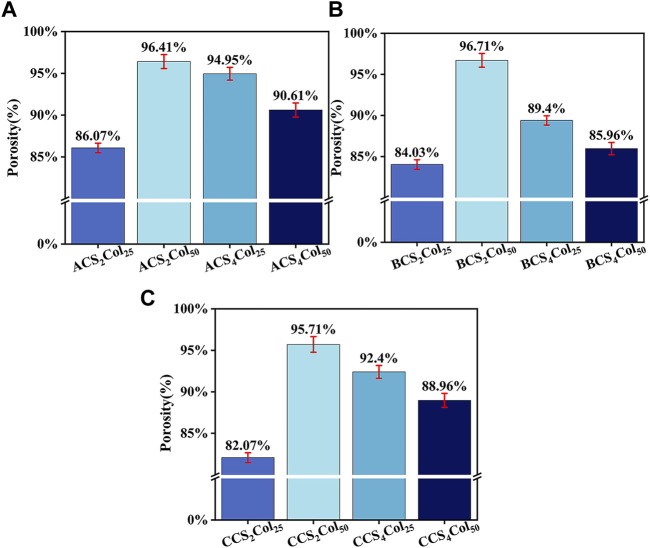
Porosity results of the scaffolds, ACSCols **(A)**, BCSCols **(B)**, and CCSCols **(C)**.

As can be seen from Figure 4, it is clear from the porosity of scaffolds of the three types of platforms that CS2Col2s as a control group has the lowest porosity, whereas CS_2_Col_50_ has the highest porosity and the lowest in the experimental group is CS_4_Col_50_. CS_2_Col_25_ should exhibit a high degree of isotropy, but as a direct freeze-dried sample its porosity is affected by the freezing speed and freezing temperature, and the overall stability and structure of the material is difficult to achieve isotropy. CS_2_Col_50_ performed very well in this porosity test, with the advantage of a lower feeding volume compared to the other two in the test group, which allowed the ice crystals to grow with less spatial resistance after phase separation. However, the consequence of this porosity is that the CS_2_Col_50_ has thinner oriented pore walls, which not only means that the walls themselves are less able to support pressure but also means that adjacent ice crystals are more likely to be connected and create more ‘defects’. As the feed volume increases, the ice crystals grow with greater spatial resistance and smaller pore size. The magnitude of the spatial resistance is not only related to the feed rate but also to the physicochemical properties of the other two phases of the feed material outside of the aqueous phase.

### Cytocompatibility of the scaffolds

The Haversian system is mostly found in the cortical bone, and low bone density or excessive defect size necessarily does not provide space for the Haversian system to exist. This results in the inability to generate tissues such as blood vessels or generating too little of them. It can be concluded from this that the self-healing ability of the organism will be dramatically increased after the generation of tissues such as blood vessels in the presence of mechanically supportive fixation devices, which is the reason why the Haversian system is present in the initial stages of bone defect repair.

In summary, if the intention is to examine whether scaffolds can function similarly to the Haversian system *in vivo* through *in vitro* cell studies, then HUVEC cells are an excellent choice.

**FIGURE 5:**
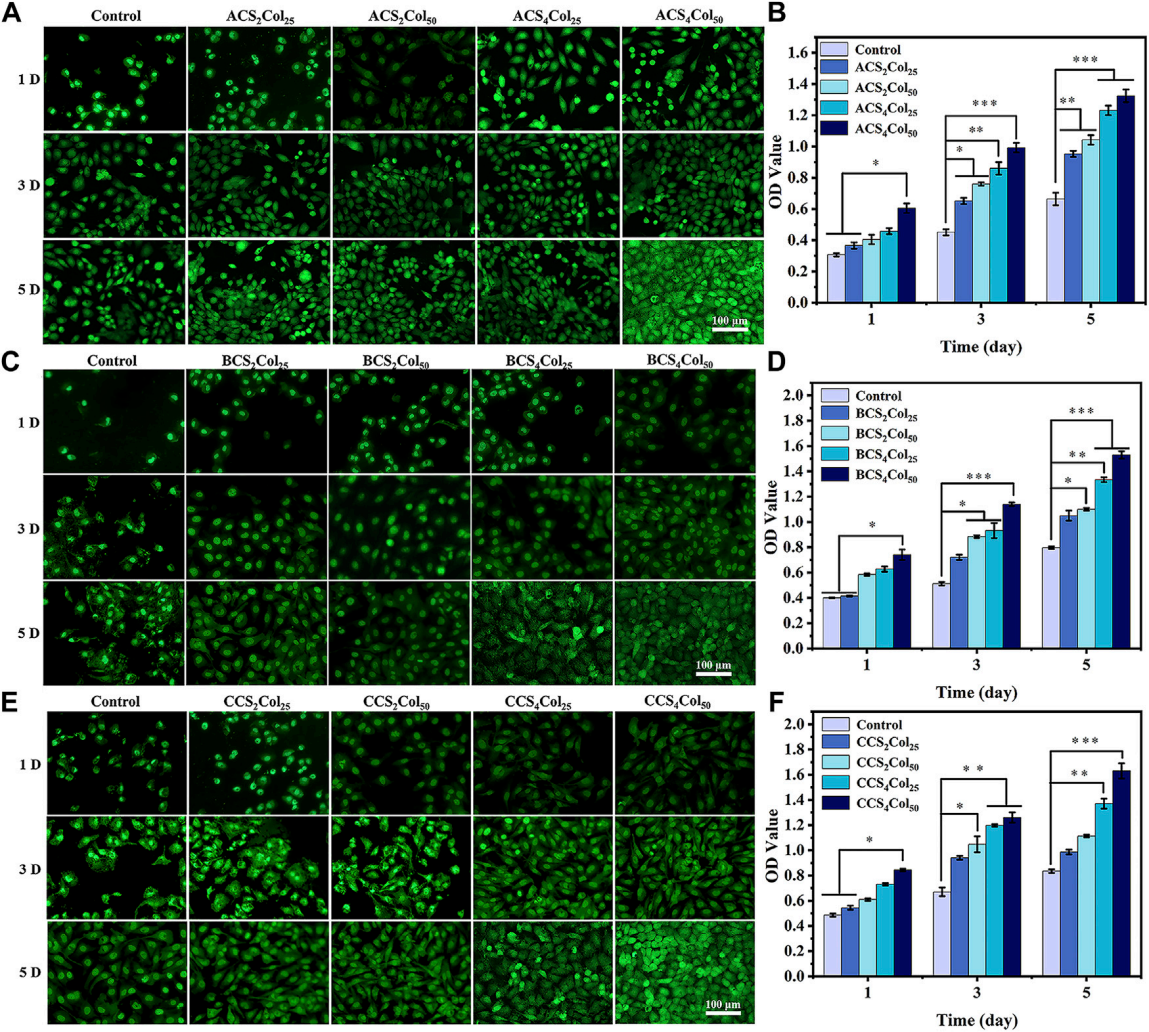
AO/EB staining **(A,C,E)** and cell proliferation activity **(B,D,F)** of the cells on the scaffolds. (*p < 0.05, **p < 0.01, ***p < 0.001) (changed the figure of BCS2Col50, 3D).

In order to investigate the cytocompatibility of the scaffolds constructed by different platforms, the growth of HUVEC cell lines after co-culture with the materials was analyzed using cell live-dead staining (AO/EB) as well as cell proliferation assays (CCK-8). The Haversian system is primarily responsible for nutrient transport and angiogenic conduits in the early stages of bone healing, and with this property in mind, umbilical vein vascular endothelial cells were used as experimental cells in the *in vitro* cytocompatibility assay of mimicking Haversian system gel scaffolds. After co-culture of HUVEC cells on the scaffolds for 1, 3 and 5 days, respectively, as shown in Figure 5, the cells density gradually increased and the cells proliferation viability was better than that of the blank control, revealing good cytocompatibility of the scaffolds. Among them, with the increase of collagen content, the pro-proliferation effect of HUVEC cells was obvious, and ACS_4_Col_50_, BCS_4_Col_50_ and CCS_4_Col_50_ were all better than other samples in the same group.

### Imaging micro-CT observation and statistical evaluation

Since all parts of the Haversian system (Haversian canal, bone tubules, etc.) play an important role in the bone repair process, and these parts cooperate with each other.

Since the prepared scaffolds mimic a complete Haversian system, the scaffolds used in the animal study should be complete in order to truly take advantage of the significant benefits of the scaffolds. Since the size of the bone defect in the model for the animal study in this study could only accommodate the scaffolds prepared by platform B, it was chosen as the scaffolds for the animal study model, and since BCS_4_Col_50_ performed better in the cell study, BCS_4_Col_50_ was finally chosen as the scaffolds for the animal study.

**FIGURE 6:**
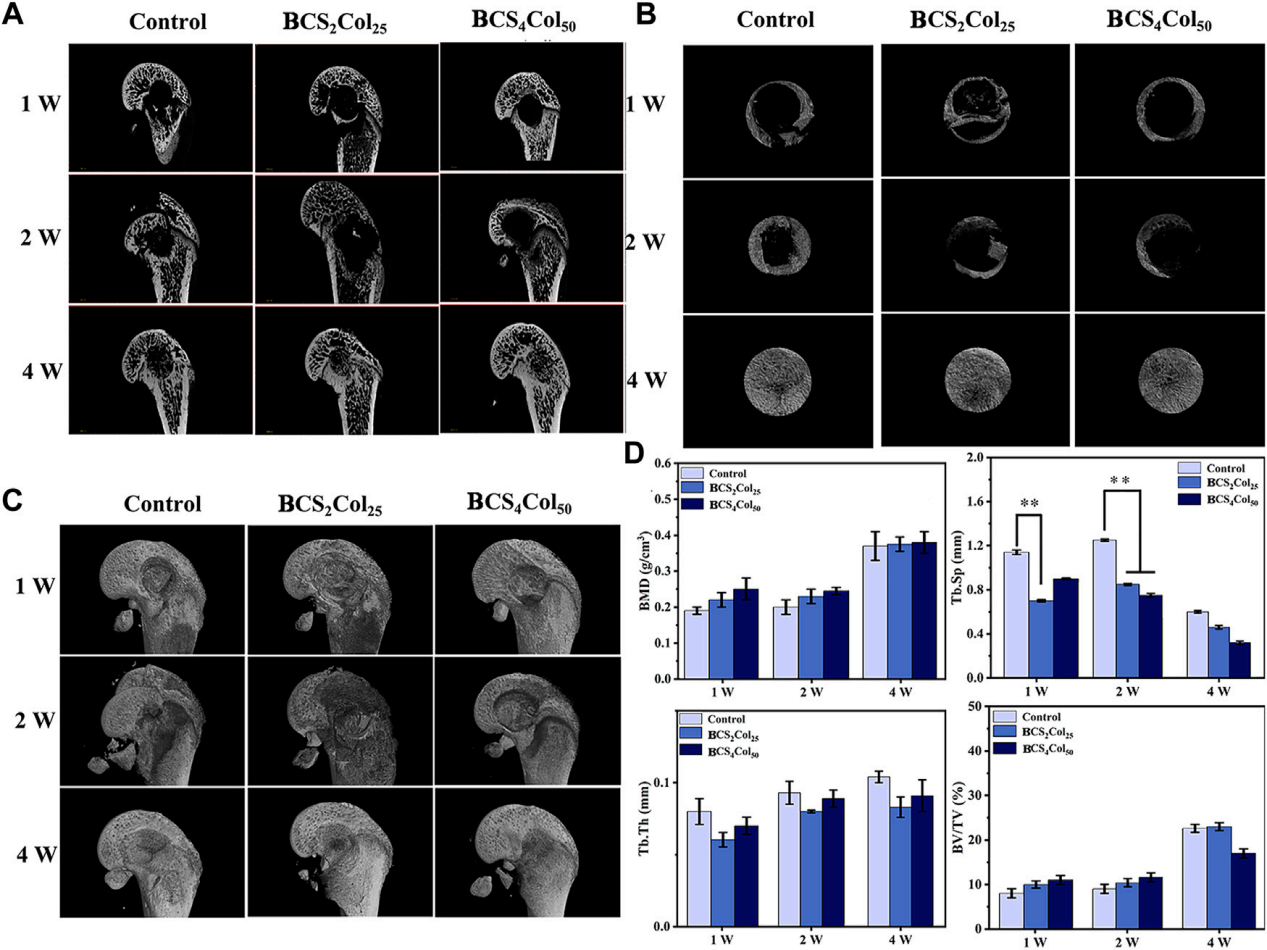
Micro-CT images of the femoral defects in rats after 1-, 2-, and 4-week operation. **(A)** Representative 2D micro-CT images. **(B)** Representative 2D micro-CT local magnification images. **(C)** Representative 3D micro-CT images. **(D)** Quantitative analysis of bone mineral density (BMD), trabecular separation (Tb.Sp), trabecular thickness (Tb.Th), and relative bone volume (BV/TV) (*p < 0.05, **p < 0.01, ***p < 0.001).

From Figure 6, 1 week after surgery, the saline blank control group showed a low density of new bone generation, and no obvious signs of bone defect repair. Both of the materials groups had new bones generation, although did not achieve bones connection of the defects. At week 2, bone crusts appeared in the defect areas of the experimental groups, demonstrating that initial connection had been achieved in the defect areas. Compared to week 1, BCS_4_Col_50_ promoted more new bones. At week 4, the blank control group had fluffy new bone structures, but both experimental groups had significant new bone generation. This may be due to the better mechanical properties of the BCS_4_Col_50_ scaffold materials, which can maintain the stability of the scaffolds more effectively and mimic the biomechanics of bone tissues to stimulate new bone regeneration through stress stimulation and postcondition. In addition, the BCS_4_Col_50_ scaffold has a rough internal surface and a large number of microchannels, which facilitates the exchange of nutrients and metabolic products within the scaffold, thus facilitating the growth of surrounding tissues and blood vessels.

The macroscopic bone repair capacity of mimicking Haversian system gel scaffolds was further assessed by statistical analysis. Bone density and relative bone volume are relatively important indicators of bone repair and their level can indicate whether the bone discontinuity has occurred. The results show that the both of the values increased with time, wherein the BCS_4_Col_50_ group was the best in the first 2 weeks. Both material groups showed a lower separation of bone trabeculae compared to the blank control group and exhibited better pro-bone repair. Bone trabeculae thickness failed to show significant changes throughout the repair process, indicating that the bone tissue did not develop severe acute inflammation leading to osteonecrosis or infected osteonecrosis as a result of the defective zone.

The reason why Haversian system mimicking scaffolds can enhance osteogenesis is that in the early stages of defect repair, the use of the bionic cavities provided by scaffolds and The good biocompatibility of scaffolds provides space for the growth of tissues such as blood vessels and uses these bionic cavities to transport nutrients and metabolic wastes, thus serving to guide the body to produce new bone and scabs.

### H&E, Masson’s and TRAP staining

**FIGURE 7:**
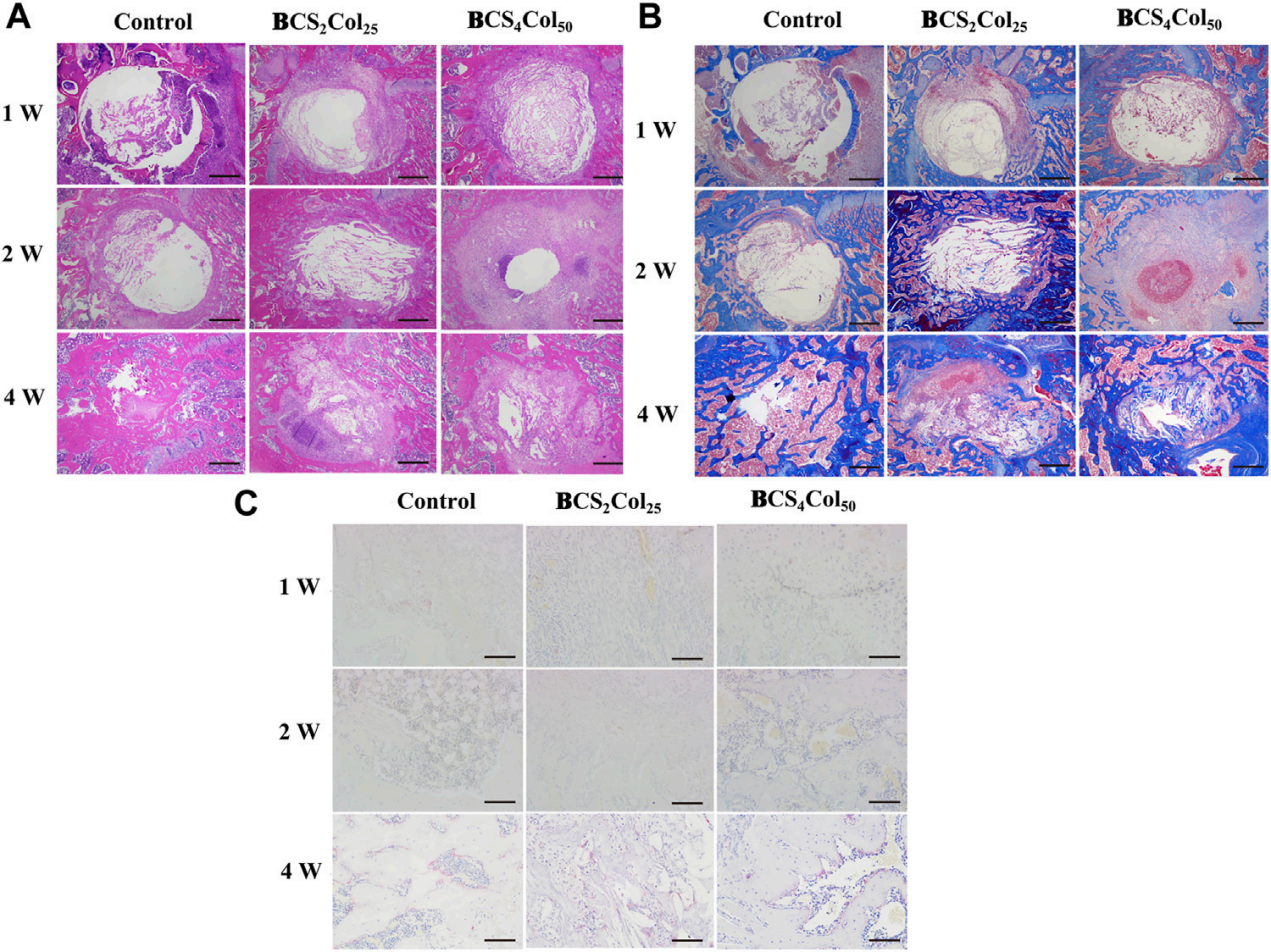
Representative images of H&E **(A)**, Masson’s **(B),** and TRAP **(C)** staining results after 1-, 2-, and 4-week operation. The scale bar of a and b is 500 μm; The scale bar of C is 100 μm.

H&E staining allows clear visualization of the nucleus and ribosomes (blue) as well as the cytoplasm and extracellular matrix (red), as shown in Figure 7A. In the first week after surgery, the cells in the blank group were disorganized within the defect, and no growth of bone trabeculae into the defect or formation of new bone tissues was visible. In the material groups, the defect area was filled with many new tissues, which was not yet tightly integrated with the surrounding bone junction, and slight fissures were still visible. The degradation of the materials was not obvious, and the Haversian system mimicking texture was visible. In addition, the BCS_4_Col_50_ caused moderate inflammation throughout the first week, indicating the initiation of the wound healing process, which is consistent with the statistical analysis of the imaging. At week 2, the BCS_4_Col_50_ group showed significantly better bone defect healing than the other groups, indicating that mimicking Haversian system gel scaffolds facilitated bone regeneration. At week 4, new bone tissue such as bone trabeculae and bone units were formed at the bone defect in the BCS_4_Col_50_ group. On the other hand, the blank group at week 1, 2 and 4 showed no significant bone crust formation and slow osteogenesis.

Masson’s staining (Figure 7B) revealed that at week 1 postoperatively, the materials was spared by cells and the material was partially degraded. At week 2 postoperatively, the BCS_4_Col_50_ group showed varying degrees of myofibrils, collagen fibers, new blue bone trabeculae and pink striated osteoid, with a large proliferation of fibroblasts, indicating a significantly greater degree of healing than the other two groups, suggesting that mimicking Haversian system gel scaffold facilitated collagen repair at the injury site.

Activation of osteoclasts is another important aspect of bone reconstruction. Upon activation, they degrade bone by secreting acid and protein hydrolases such as tissue proteinase K (CTSK), and then dissolve collagen and other matrix proteins produced during bone resorption to absorb aged and damaged bone. However, excessive osteoclasts can lead to bone resorption, so the process of bone reconstruction is a dynamic balance between osteogenesis and osteolysis. The amount of osteoclasts and their status were observed by TRAP staining at the site of bone damage. In the BCS_4_Col_50_ group, osteoclast was significantly less than the blank group and the CS_2_Col_25_ group at week 4, indicating that the scaffolds were beneficial to bone reconstruction.

### Immunofluorescence staining

**FIGURE 8:**
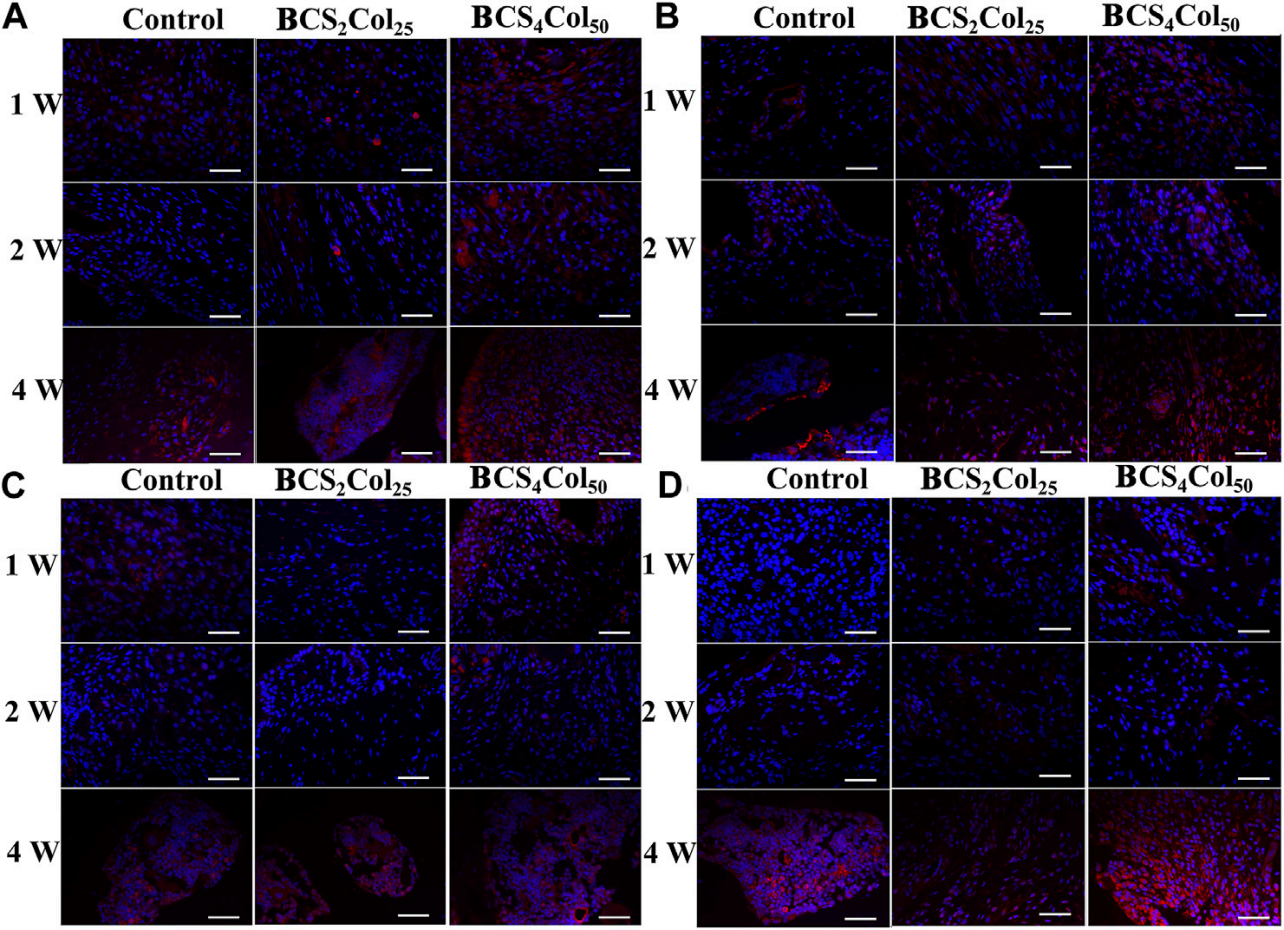
Representative immunofluorescence images of the femoral defects in rats at week 1, 2, and 4 postoperatively. **(A)** Col, **(B)** OCN, **(C)** OPN, and **(D)** Runx 2. Scale bar is 50 μm.

Immunofluorescence staining of osteocalcin (OCN), osteopontin (OPN), type I collagen (Col-1), osteogenic transcription factor (Runx 2) was utilized to analyze the production of new bone at the site of bone injury, as shown in Figure 8. At week 1, 2 and 4 after surgery, the expression of Col, OCN, OPN and Runx 2 was significantly increased with time. In addition, the expression intensity of the BCS_4_Col_50_ group was the highest, higher than that in the CS_2_Col_25_ group, indicating that the constructed mimicking Haversian system structure had a significant bone-enabling effect.

### Immunohistochemical staining

**FIGURE 9:**
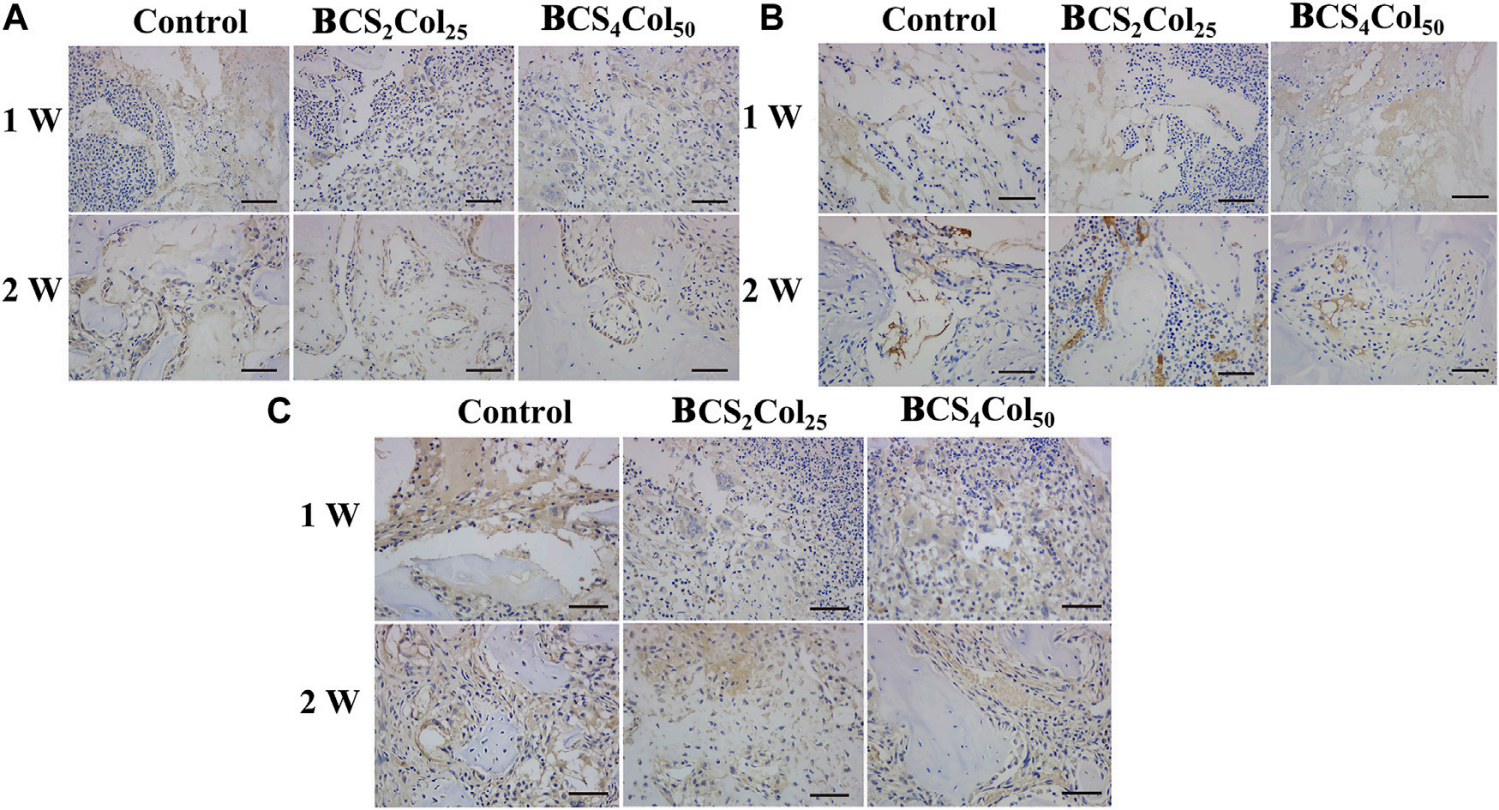
Representative immunohistochemical images of the femoral defects in rats at week 1 and week 2 postoperatively. **(A)** IL-1β, **(B)** IL-6, and **(C)** TNF-α.The scale bar is 50 μm. (changed the figure of IL-1β, BCS2Col25, 2w).

Interleukin-6 (IL-6), tumor necrosis factor (TNF-α) and Interleukin-1β (IL-1β) are typical pro-inflammation related factors. From the immunohistochemical results (Figure 9), it was observed that the expression of the factors at the site of bone injury in both of the material groups was significantly lower than that in the blank control group at week 1 and 2 postoperatively, indicating that the materials had anti-inflammatory properties to some extent. What’s more, the intensity of the expression in the CS_4_Col_50_ experimental group was significantly lower than that in the other two groups, indicating that mimicking Haversian system gel scaffolds maybe reduce the occurrence of inflammation through regulation of the microenvironment or new tissue regeneration.

## Conclusion

The main innovation of this study is, first, to mimic the structure of the Haversian system formed in the early stage of bone tissue healing and to construct a universal preparation platform based on the directional freezing technology, which can be used to prepare scaffolds with cavities roughly arranged at right angles to each other; second, to use the directional freezing technology to prepare chitosan/collagen mimicking Haversian system gel scaffolds. Physiochemistry, cells, and animal experiments showed that Haversian system mimicking scaffolds possess an anisotropic structure and can effectively accelerate bone regeneration to heal of bone defects.

## Data Availability

The original contributions presented in the study are included in the article/Supplementary Material; further inquiries can be directed to the corresponding author.
