# Supplementary material for: Freeze-drying platforms design for batch fabrication of Haversian system mimicking scaffolds with enhanced osteogenesis
Source: Front Bioeng Biotechnol. 2022 Oct 11;10:1013528. doi: 10.3389/fbioe.2022.1013528 (PMC9593081; doi:10.3389/fbioe.2022.1013528)
Supplement: Supplementary file 1 [file Table1.DOCX]

***Supplementary Material***

Support Table S1 Thermal conductivity components a results of various tests

| Physical indicators | Standard values | The readings |
| --- | --- | --- |
| Breaking load(gf) | >4 | 6.46 |
| Elongation(%) | 8-16 | 8.78 |
| Hardness(Hv) | 58-62 | 59.58 |
| A fuse(A，10mm) | 0.36 | 0.370 |
| thermal conductivity(W/(m·K)) | 377 | 356 |
| Cu | ≥99.95 | >99.99 |
| Others | <0.05 | <0.01 |

Support Table S2 Test results of thermal insulation components a

|  | Standard values | Detected values (average) |
| --- | --- | --- |
| Thermal conductivityW/(m·K) | ≤0.024 | 0.021 |
| Compressive strength or 10% compressive stress (kPa) | ≥150 | 236 |
| Dimensional stability (30℃，%) | 1.0 | 1.0 |
| Closing ratio（%） | ≥90 | 96 |
| Water absorption（%） | ≤4 | 2.76 |


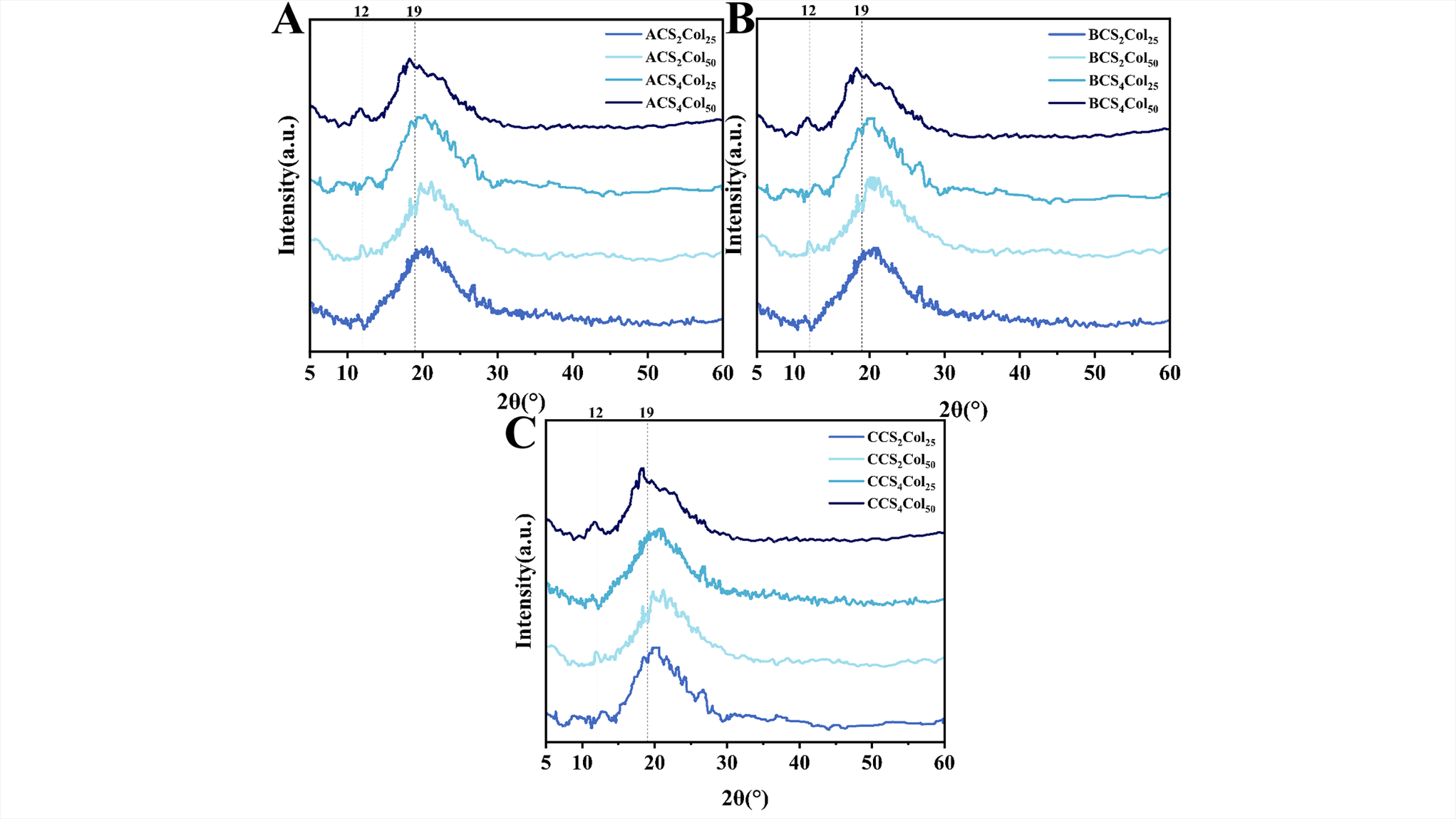


Support figure S1 Platform A,B,C gel scaffold XRD(A) FT-IR of platform A ACSCol (B) XRD of platform B BCSCol (C) XRD of platform C CCSCol

As shown in figure S1, the peak intensity of the CSCol 7.5° peak increases with increasing Col addition, and the broad 20.5° peak appears as a double peak in the group with the highest Col content at 17.5° and 20.5° buns, indicating that the peak intensity of Col is positively correlated with the addition ratio and amount after platform processing. On the one hand, this indicates a better crystallinity of Col, and on the other hand, it shows that both CS and Col are still well bound at high levels. The intermolecular rigidity of the remaining two phases is weakened by the bi-directional squeezing pressure that occurs during the growth of the ice crystal due to the binding of the platform bin, and the interaction between the CS and Col molecules also affects the CS crystallinity to some extent, while the Col intermolecular interaction is weakly affected. This is probably due to the weak peak intensity at low concentrations and the resulting fusion peak.


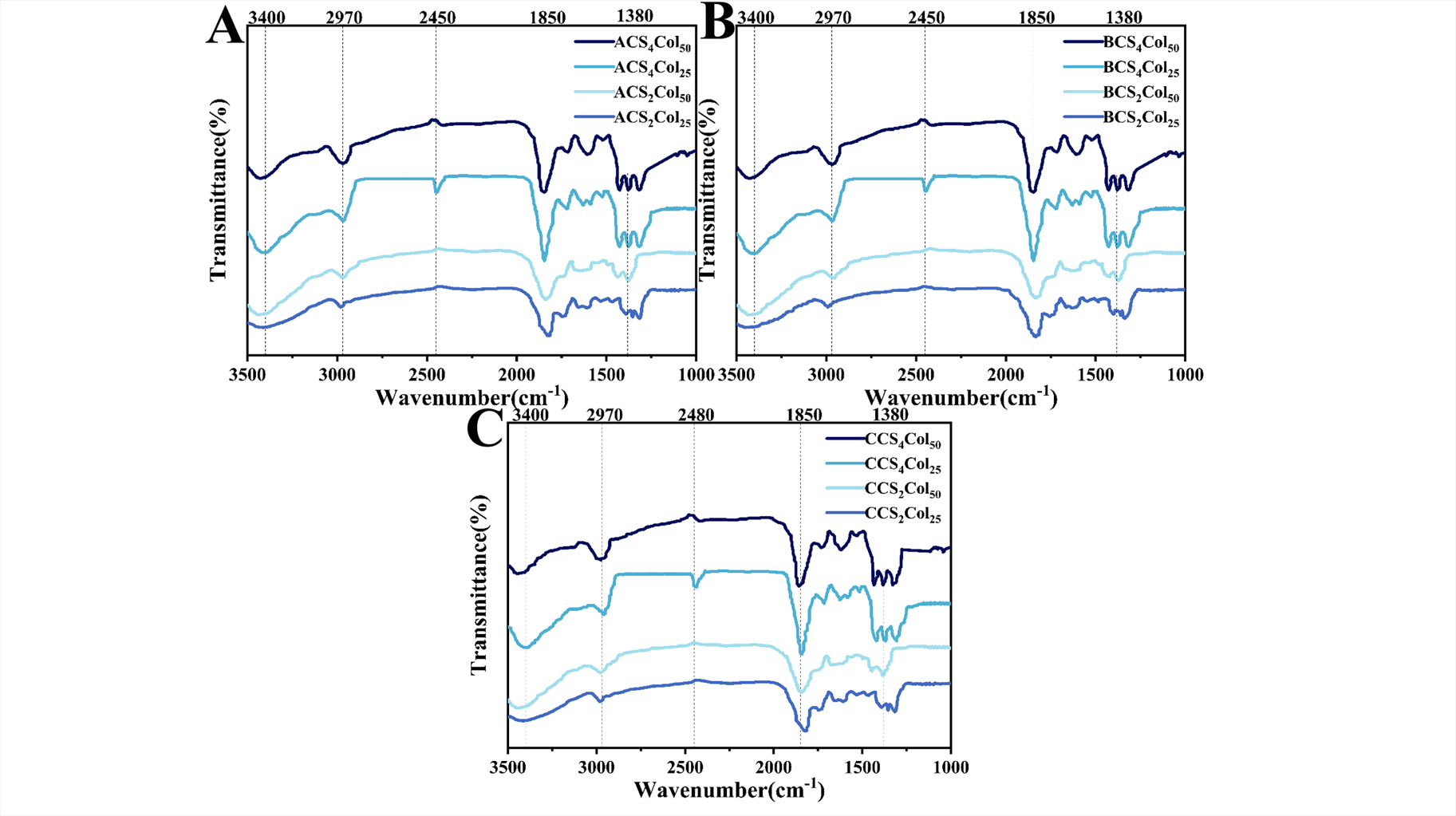


Support figure S2 Platform A,B,C gel scaffold FT-IR(A) FT-IR of platform A ACSCol (B) FT-IR of platform B BCSCol (C) FT-IR of platform C CCSCol

As shown in figure S2, the characteristic absorption peaks in the amide I, II and III bands of the CSCol gels occur around 1850 cm-1, 1600 cm^-1^ and 1380 cm^-1^. The peaks near the amide III band are notable for being located around 1300 cm-1 and are positively correlated in intensity with the amount of CS fed. carboxyl, amino and amide bonds are present in both Col and CS molecules, the difference being the variety of peptide bonds in Col. The large peak around 3400 cm^-1^ contains hydroxyl, amino, and N-H vibrational absorptions on the amide bond. This is due to the abundance of hydroxyl, amino and acetylamino groups in the chitosan molecule, which are active groups that not only make the peaks stronger but also cause the CS to bind more tightly to the Col. The same is true for the amide I band around 1850 cm^-1^, where the intensity of the peak increases significantly with increasing chitosan content, but there is a significant blue shift in the amide I band, which also occurs in the prepared collagen, where the blue shift decreases with increasing chitosan content but is still around 100 cm^-1^ away from the standard amide I band. The intensity of the amide II band also changes with increasing CS content, but its intensity is related to both components, with CS_2_Col_25_ being more intense than the amide II band of CS_2_Col_50_ but both significantly less intense than the amide II bands of CS_4_Col_25_, CS_4_Col_50_, and the same occurs with the amide II bands of CS_4_Col_25,_ CS_4_Col_50_ This indicates that the CS content significantly strengthens the peak intensity, but the Col content suppresses it. The weakening of the peak intensity indicates that the N-H bending vibration and the C-N stretching vibration are suppressed, which indicates that as the concentration of both increases, new hydrogen bonds emerge, and the increase in concentration does not affect the binding The tightness of the bonding. In support of this, the two peaks near the 1380 cm^-1^ amide III band show a significant increase in peak intensity with increasing levels of both, and the stronger absorption peak as a result of the saturated C-H deformation vibration indicates the emergence of a new bonding mode with increasing levels. Also in support, the peak near 2970 cm^-1^ is the multiplication peak of the amide II band, which increases in intensity with increasing CS and Col. This increase in intensity usually indicates that the amide bond has been affected and a conformational change has occurred. The results show that the Col and CS molecules of the bionic gel interact strongly by means of hydrogen and ionic bonds, and that hydrogen bonds are easily formed between the carbonyl, hydroxyl and amino groups possessed by Col and the hydroxyl and amino groups possessed by CS, which are given the environment by the great pressure during the growth of the ice crystals due to the closure of the moulded sample grooves. In addition the isoelectric point (pl) of collagen is consistent with most proteins in the human body being less than 6, depending on the raw material and extraction process its isoelectric point is approximately 4.8-5.2, so under neutral conditions as a cationic CS can also form ionic bonds with the negatively charged Col. This bonding ability results in the formation of a polycation-polyanion complex within the gel. In contrast, the results for the control group CS_2_Col_25_ can be clearly seen as the strength of the groups is generally weak and the bonding and influence on each other is not outstanding.

In contrast, a significant decrease in the characteristic peaks of BCS_2_Col_25_, BCS_2_Col_50_, BCS_4_Col_25_, BCS_4_Col_50_ and CCS_2_Col_25_, CCS_2_Col_50_, CCS_4_Col_25_, CCS4Col50 at around 1850 cm^-1^ was found for platforms B and C compared to platform A. The decrease in the characteristic peak of amide I can be attributed to the interaction between CS and Col. In addition, there is a certain "blue shift" in the position of the peak at 1380 cm^-1^, which we believe is due to the difference in material morphology caused by the different platforms, which in turn affects the bonding and the strength of the hydrogen bonds, resulting in a shift in the characteristic peak.

Compared to the platform C, the platform A, B materials show a clear 'blue shift' at around 1043 cm^-1^, which is due to structural and other reasons, while the change in the strength of the peak at 1600 cm-1 is due to the influence of hydrogen bonding.


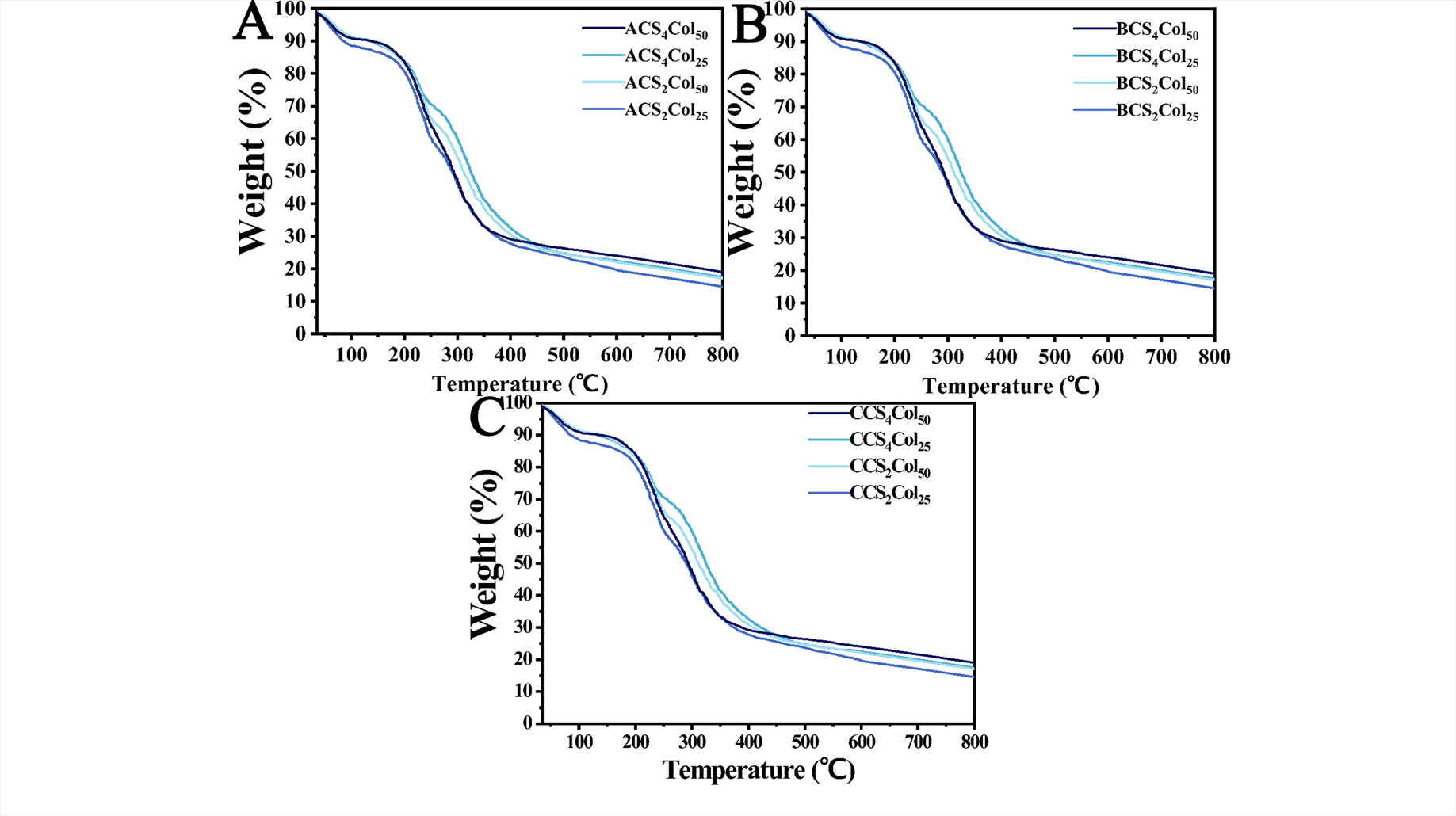


Support figure S3 Platform A,B,C gel scaffold TG(A) TG of platform A ACSCol (B) TG of platform B BCSCol (C) TG of platform C CCSCol

Both the raw material, control and oriented frozen gel scaffolds underwent a process of dehydration and weight loss in the initial phase, followed by decomposition in the second phase, followed by inactivation and deconstruction in the shorter temperature interval of Col. Both components then underwent a violent long chain decomposition to water and gas, leaving a small amount of organic matter behind. Comparing the samples prepared on the models of the three types of platforms, there was a slight decrease in the thermal stability of the CS_2_Col_25_ compared to the CSCol gel scaffold in general agreement. The scaffolds all showed different stages of heat uptake during the weight loss process. This may be due to the enhanced intermolecular interactions during the extrusion process during ice crystal growth.

As can be seen from the graphs, the decomposition rates of platforms B and C slow down significantly in the second stage compared to platform A. This may be due to structural differences that lead to changes between the CS and Col molecules, which in turn affect the thermal stability of the material.

The decomposition temperature at the start of the second stage was lower in platforms A and C compared to platform B, and the final weight loss rate was also lower, suggesting that there are differences in the synthesis of the materials at different platforms and that this can lead to changes in thermal stability.

Compared to platforms A and B, platform C shows a decrease in the initial decomposition temperature and a significant slowdown in the rate of decomposition during the second phase, which may be attributed to material interactions; and a lower final weight loss rate for platform C.
